# Genetic Algorithm-Based Optimization of Curved-Tube Nozzle Parameters for Rotating Spinning

**DOI:** 10.3389/fbioe.2021.781614

**Published:** 2021-12-03

**Authors:** Wenhui Li, Kang Liu, Qinghua Guo, Zhiming Zhang, Qiaoling Ji, Zijun Wu

**Affiliations:** ^1^ Hubei Digital Textile Equipment Key Laboratory, Wuhan Textile University, Wuhan, China; ^2^ School of Mechanical Engineering and Automation, Wuhan Textile University, Wuhan, China

**Keywords:** genetic algorithms, optimization, rotating spinning, curved-tube nozzle, nanofibers

## Abstract

This paper proposes an optimization paradigm for structure design of curved-tube nozzle based on genetic algorithm. First, the mathematical model is established to reveal the functional relationship between outlet power and the nozzle structure parameters. Second, genetic algorithms transform the optimization process of curved-tube nozzle into natural evolution and selection. It is found that curved-tube nozzle with bending angle of 10.8°, nozzle diameter of 0.5 mm, and curvature radius of 8 mm yields maximum outlet power. Finally, we compare the optimal result with simulations and experiments of the rotating spinning. It is found that optimized curved-tube nozzle can improve flow field distribution and reduce the jet instability, which is critical to obtain high-quality nanofibers.

## Introduction

**FIGURE 1:**
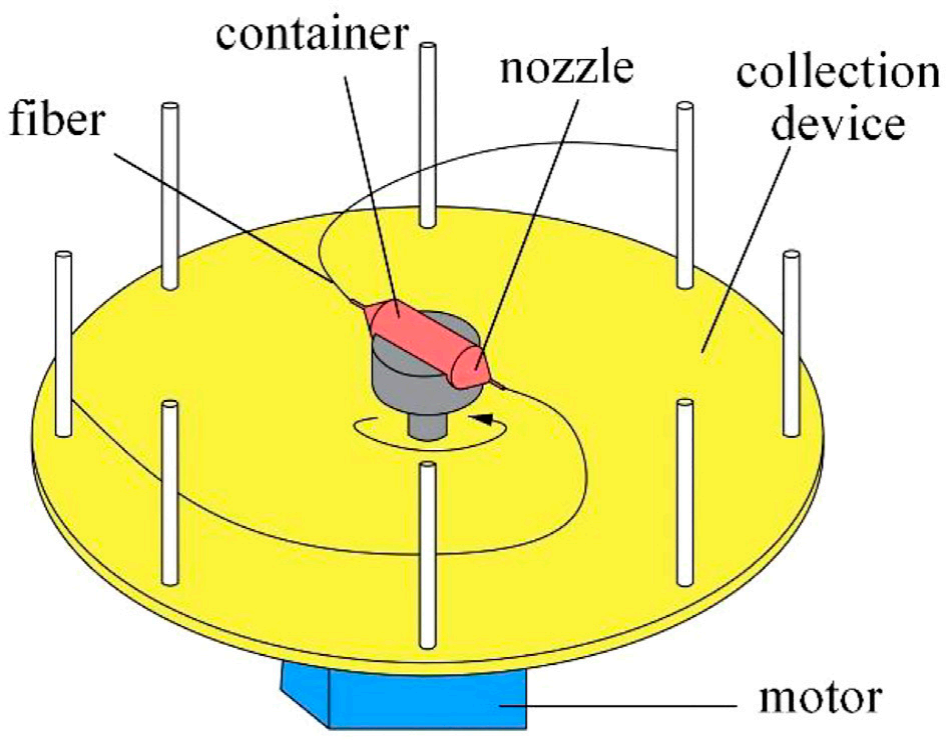
Rotating spinning system.

Nanofibers(Vasita and Katti, 2006; Bera, 2017; Kenry and Lim, 2017; Nayak, et al., 2011) have a wide range of applications in emerging areas such as energy generation (Persano, et al., 2015), water treatment (Saud, et al., 2015), healthcare (Feng, et al., 2019), and biomedical engineering (Xie, et al., 2008) owing to their excellent physicochemical properties and characteristics. Rotating spinning (Zhang and Lu, 2014) is an emerging method for nanofiber preparation. Figure 1 shows a basic rotating spinning setup including a container, two nozzles, a motor, and a collection device. In the process of rotating spinning, polymeric solution is ejected from the nozzle outlet, and jet is stretched by the centrifugal force to form solidified nanofibers. Rotating spinning overcomes limitations of materials and enables nanofiber production at lower cost. Therefore, it possesses the commercial production potential of nanofibers, compared with traditional nanofiber synthesis strategies such as self-assembly (Whitesides and Grzybowski, 2002), melt (Wei, 2018), phase separation (Ma and Zhang, 1999), template synthesis (Wade and Wegrowe, 2005), stretching (Ondarcuhu and Joachim, 1998), and electrospinning (Bognitzki, et al., 2001; Hohman, et al., 2001; Subbiah, et al., 2005; Teo and Ramakrishna, 2006; Badieyan and Janmaleki, 2015; Deshawar and Chokshi, 2017; Chen, et al., 2021). Therefore, more and more attention has been paid to rotating spinning technology.

The previous researches mainly focused on mechanisms of rotating spinning. Noroozi et al. (2020) established the string model to study the behavior of viscous jet in the rotating spinning. Divvela et al. (2017) developed a discrete model to predict the rotating trajectory of the viscous jet in rotating spinning. Padron et al. (2013) used high-speed photography to capture the forming process of the initial jet. Riahi (2017) established the mathematical model of rotating spinning and carried out the jet stability analysis.

Based on mechanisms of the rotating spinning, there are many studies about the nozzle structure. Xu et al. (2014) compared the nozzle and nozzle-less rotating spinning process. The results showed that the nozzle-type spinning could be easier to obtain thicker nanofibers. Chen et al. (2020) discussed the effect of nozzle tube length on jet stability through the simulation of the solution motion in the rotating spinning nozzle. Lu et al. (2013) controlled the diameter distribution of nanofibers by changing the nozzle diameter. Zhmayev et al. (2015) explored the influence of nozzle direction on the initial jet motion. Lai et al. (2021) proposed four types of nozzle structures: stepped, conical straight, conical, and curved tube. Simulations and experiments of the rotating spinning showed that the curved-tube nozzle is the optimal.

**FIGURE 2:**
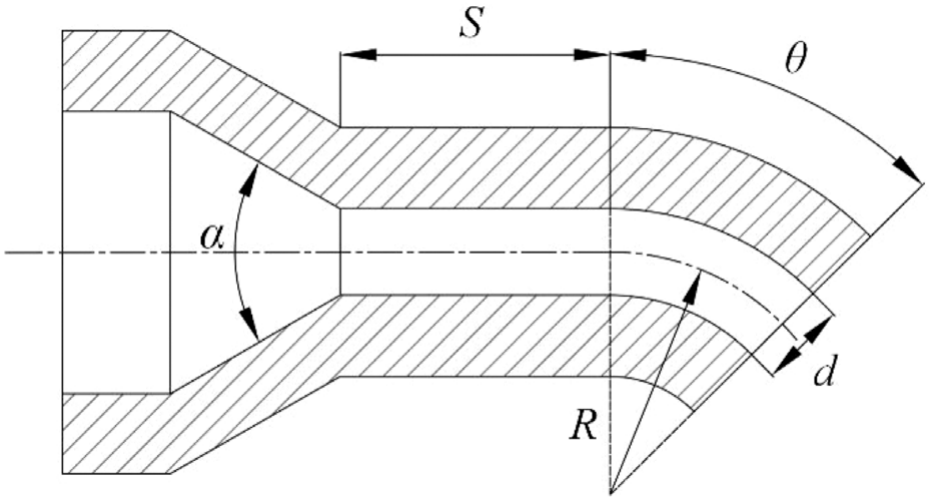
The structure of curved-tube nozzle.

Nozzle has become the key part of the rotating spinning equipment affecting the solution motion state, the jet tensile motion, and the morphology of nanofibers. The structure of curved-tube nozzle is shown in Figure 2; parameters contain bending angle *θ*, straight tube length *S*, curvature radius *R*, nozzle diameter *d*, and taper α. Much progress has been achieved currently in the study of rotating spinning mechanism. However, the optimization of nozzle structure is still in a stage of infancy. In this paper, an optimization approach for structure design of curved-tube nozzle is developed, wherein the outlet power obtained by the product of outlet velocity and force is employed as the objective function, and genetic algorithm is applied to find the optimum combination of nozzle structure parameters.

Genetic algorithm is a highly parallel, random, and adaptive global optimization search algorithm, which was studied by Professor Holland in the 1960s and improved by Dejon and Goldberg to form genetic algorithm (Katoch, et al., 2020). The strong versatility and global convergence can avoid the optimization process falling into local optimal solution (Kumar, et al., 2010; Bhoskar, et al., 2015). Therefore, it is very suitable for multivariate optimization problem (Wiśniewski, 2004; Guo, et al., 2009; D’Addona and Teti, 2013; Asadi, et al., 2014; Jiang, et al., 2021).

This paper mainly consists of three parts. In the first part, the correlation between the nanofiber morphology and structure parameter is established using dynamic model of rotating spinning. Outlet power of polymer solution is proposed as optimization objective. In the second part, genetic algorithms have been used to search for the optimal solution in a reasonable range. The numerical simulation of fluid motion in different curved-tube nozzles has been proceeded to analyze the distribution of flow field. In the third part, rotating spinning experiments have been carried out by straight-tube nozzle and curved-tube nozzle, respectively. We compared the simulation.

The variables and parameters used in this article are shown in Table 1 and Table 2 respectively.

**TABLE 1:** The variables used in the formula and models

Variables	Description
Θ	Bending angle
*R*	Curvature radius
*D*	Nozzle diameter
*u*	Relative velocity

**TABLE 2:** The parameters used in the formula and models

Parameters	Description
*P*	Stress tensor
*T*	Partial stress tensor
*R*	Position vector
*F*_k_	Coriolis force
*F*_C_	Centrifugal force
*D*	Strain rate tensor
*I*_2_	Invariant of the strain rate tensor
*k*	Consistency index
*n*	Rheological index
*D*	Container diameter
*L*	Container length
ω	Angular velocity
α	Nozzle taper
*S*	Straight tube length
ρ	Density

## Flow Model of Spinning Solution In Curved-Tube Nozzle

### Hydrodynamics Analysis for Rotating Spinning

**FIGURE 3:**
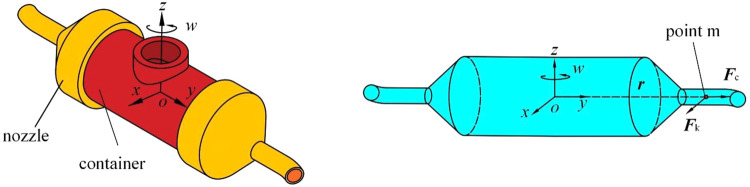
Motion model of rotating spinning. **(A)** The structure of curved-tube nozzle and container. **(B)** The spinning solution in curved-tube nozzle and container results with the experimental observations of power-law fluid to validate the optimization model.

The model of rotating spinning is shown in Figure 3. Cartesian coordinate system *oxyz* is stationary relative to rotating container. This non-inertial coordinated system rotates around the axis oy at angular velocity. The origin *o* is at the center of rotation. The axis *oz* coincides with the nozzle axis.

In the process of rotating spinning, the flow solution is subject to pressure, centrifugal force, Coriolis force, viscous force, and gravity. The fluid motion can be regarded as steady motion. The continuity equation and momentum equation in the rotating frame are given as

$$\begin{array}{l} \nabla \cdot U=0\\ U\cdot \nabla U=\frac{1}{\rho }\nabla \cdot P-w\times \left(w\times r\right)-2w\times U \end{array}$$ (1)

where ***U*** is relative velocity vector, *p* is stress tensor, *p* = −*p* + ***T***, ***T*** is partial stress tensor, *p* is pressure, *w* is angular velocity, ρ is density of the solution, ***r*** is position vector, ***w*** × (***w*** × ***r***) is centrifugal force, and 2 ***w*** × ***U*** is Coriolis force.

Because the solution used in rotating spinning experiment is power-law fluid, the constitutive equation can be written as

$$\begin{array}{l} T=2k{\left|I_{2}\right|}^{\frac{n-1}{2}}D\\ I_{2}=2tr\left(D^{2}\right) \end{array}$$ (2)

where *k* is consistency index, *n* is the rheological index, *D* is the strain rate tensor, and *I*_2_ is the invariant of the strain rate tensor.

### Formula Derivation of Outlet Power

**FIGURE 4:**
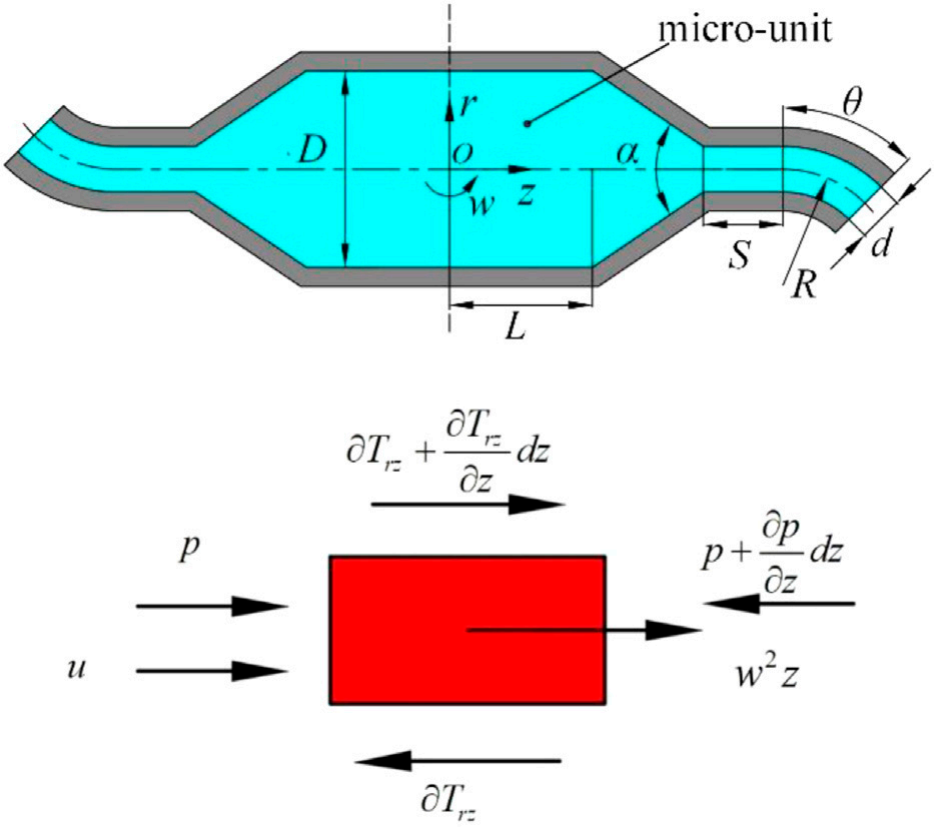
Rotating spinning model. **(A)** One-dimensional flow of spinning solution. **(B)** Force diagram of micro-unit.

To obtain analytical solution, we simplify the fluid motion in the nozzle to one-dimensional laminar flow. The fluid motion on plane *orz* is shown as Figure 4A.

The Coriolis force can be ignored because it is counteracted by the pressure gradient along *r* axis of spinning solution in the container and nozzle tube. Figure 4B shows the forces acting on the micro-unit obtained from the flow field. Because of the existence of free flow surface in the container, the pressure gradient along *z* axis can be ignored. Momentum Eq. 1 can be given as follows:

$$\rho w^{2}z=\frac{\partial T_{rz}}{\partial r}$$ (3)

where *z* is the axial position, *w* is angular velocity, and *ρ* is density of the solution.

The power-law fluid flows along the axis *z* direction. The constitutive Eq. 2 is simplified as follows:

$$T_{rz}=k{\left(\frac{du}{dr}\right)}^{n}$$ (4)

where *u* is the flow velocity of spinning solution, *r* is the radial position, and *k* and *n* are the rheological indexes.

In addition, the following boundary conditions at the container wall should to be satisfied as

$$z=0,u=0;r=\frac{D}{2},u=0$$ (5)

where *D* is the container diameter.

Substituting Eq. 4 into Eq. 3 and combined with Eq. 5, flow field distribution in the container is deduced as follows:

$$u_{1}=\frac{n}{n+1}{\left(\frac{\rho \omega^{2}z}{k}\right)}^{\frac{1}{n}}\left({\left(\frac{D}{2}\right)}^{\frac{n+1}{n}}-r^{\frac{n+1}{n}}\right)$$ (6)

The average flow velocity of spinning solution in the container is expressed as

$$u_{1a}=\frac{n}{1+3n}{\left[\frac{\rho \omega^{2}z}{k}\right]}^{\frac{1}{n}}{\left(\frac{D}{2}\right)}^{\frac{n+1}{n}}$$ (7)

**FIGURE 5:**
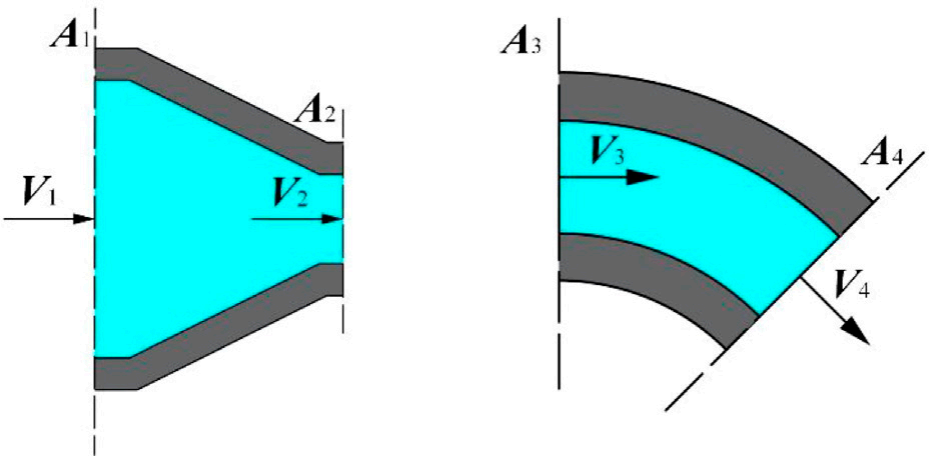
Control body of the curved-tube nozzle. **(A)** Control body of shrinkage tube. **(B)** Control body of bend tube.

As shown in Figure 5A, the container outlet, nozzle inlet, and pipe wall are taken as the control bodies. According to Eq. 7, the average velocity of container outlet *V*_1_ is calculated as

$$V_{1}=\frac{n}{1+3n}{\left[\frac{\rho \omega^{2}L}{k}\right]}^{\frac{1}{n}}{\left(\frac{D}{2}\right)}^{\frac{n+1}{n}}$$ (8)

where *L* is the distance from the container outlet to the rotation center.

Based on the mass conservation equation of steady flow *V*_1_ *A*_1_ = *V*_2_ *A*_2_, the average velocity in straight pipe inlet *V*_2_ can be written as

$$V_{2}=\frac{n}{d^{2}\left(1+3n\right)}{\left[\frac{\rho \omega^{2}L}{k}\right]}^{\frac{1}{n}}{\left(\frac{D}{2}\right)}^{\frac{3n+1}{n}}$$ (9)

In the nozzle tube, there is a pressure gradient along the axial direction. Momentum equation can be simplified as

$$\rho w^{2}z+\frac{\partial p}{\partial z}=\frac{\partial T_{rz}}{\partial r}$$ (10)

The boundary conditions at the nozzle inlet and the wall can hold as

$$z=L+\frac{D-d}{2}\cot\frac{\alpha }{2},u_{2a}=V_{2};r=\frac{d}{2},u=0$$ (11)

where *α* is taper and *d* is the nozzle diameter.

Substituting Eq. 4 into Eq. 10 and combined with boundary conditions (11), the distribution of flow field in the straight tube of nozzle is obtained as follows:

$$u_{2}=\frac{n}{1+n}{\left[\frac{\rho \omega^{2}z+c}{k}\right]}^{\frac{1}{n}}\left({\left(\frac{d}{2}\right)}^{\frac{n+1}{n}}-r^{\frac{n+1}{n}}\right)$$ (12)

The average velocity is given as

$$u_{2a}=\frac{n}{1+3n}{\left[\frac{\rho \omega^{2}z+c}{k}\right]}^{\frac{1}{n}}{\left(\frac{d}{2}\right)}^{\frac{n+1}{n}}$$ (13)

where *c* is pressure drop. According to the boundary condition (11), it can be given as follows:

$$c=\frac{\partial p}{\partial z}=\rho \omega^{2}\left[\left({\left(\frac{D}{d}\right)}^{3n+1}-1\right)L-\frac{D-d}{2}\cot\frac{\alpha }{2}\right]$$ (14)

It is found that the pressure drop is related to the rheological parameters of solution, rotation angular velocity, and nozzle structure.

The nozzle straight tube outlet and nozzle elbow outlet are taken as the control bodies, as shown in Figure 5B, and the average velocity at the nozzle straight tube outlet *V*_3_ is expressed as follows:

$$V_{3}=\frac{n}{1+3n}{\left[\frac{\rho \omega^{2}}{k}\left({\left(\frac{D}{d}\right)}^{3n+1}L+S\right)\right]}^{\frac{1}{n}}{\left(\frac{d}{2}\right)}^{\frac{n+1}{n}}$$ (15)

According to the mass conservation of the steady flow, the average velocity at the nozzle outlet ***V***_4_, the Coriolis force ***F***_k_, and centrifugal force ***F***_C_ on the jet are expressed as follows:

$$\left\{ \begin{array}{l} V_{4}=V_{3}\cos\theta i+V_{3}\sin\theta j\\ F_{K}=2\omega V_{3}\left(-\sin\theta i+\cos\theta j\right)\\ F_{C}=\omega^{2}\left(L+\frac{D-d}{2}\cot\frac{\alpha }{2}+S+R\sin\theta \right)i\\ +\omega^{2}R\left(1-\cos\theta \right)j \end{array}\right.$$ (16)

Therefore, the power at the nozzle outlet can be written as

$$\begin{array}{l} P=\frac{\omega^{2}n}{1+3n}{\left[\frac{\rho \omega^{2}}{k}\left({\left(\frac{D}{d}\right)}^{3n+1}L+S\right)\right]}^{\frac{1}{n}}{\left(\frac{d}{2}\right)}^{\frac{n+1}{n}}\\ \begin{array}{ccc} & & \cdot \end{array}\left[\left(L+\frac{D-d}{2}\cot\frac{\alpha }{2}+S\right)\cos\theta +R\sin\theta \right] \end{array}$$ (17)

## Process of Optimization for Curved-Tube Nozzle

### Optimization Model for Structure Parameters of Curved-Tube Nozzle

The main parameters of the curved-tube nozzle are *θ*, *S*, *R*, *d*, and *α*. Bending angle *θ*, curvature radius *R*, and nozzle diameter *d* are selected as the optimized design variables considering the influence of various parameters on the fluid motion during the spinning process. According to actual spinning conditions, other parameters are set as constants, and the optimization objective function is established as follows:

maxf(x)=maxP(x) (18)

The design variables of the model can be written as

$$x={\left\{\begin{array}{c} \mathrm{bending}\mathrm{angle}\theta \\ \mathrm{curvature}radiusR\\ \;\mathrm{nozzle}diameterd \end{array}\right\}}^{T}$$ (19)

The system parameters of the optimization model are shown in Table 3.

**TABLE 3:** System parameters of optimizing model

Item	Parameters	Description	Value
Optimization object	*p*	Outlet power	Max
Design parameters	Θ	Bending angle	(0, 90)
	*R*	Curvature radius	(3, 8)
	*d*	Nozzle diameters	(0.5, 1)
Other parameters	*K*	Consistency index	15.3
	*n*	Rheological index	0.464
	*D*	Container diameter	10
	*L*	Container length	30
	ω	Angular velocity	4,000
	α	Nozzle taper	90
	*S*	Straight tube length	5
	ρ	Density	1,000

The simplified fitness function can be written as

$$f\left(x\right)=d^{-2}\left[\left(40-\frac{d}{2}\right)\cos\theta +R\sin\theta \right]$$ (20)

### Application of Genetic Algorithm in Curved-Tube Nozzle Optimization

**FIGURE 6:**
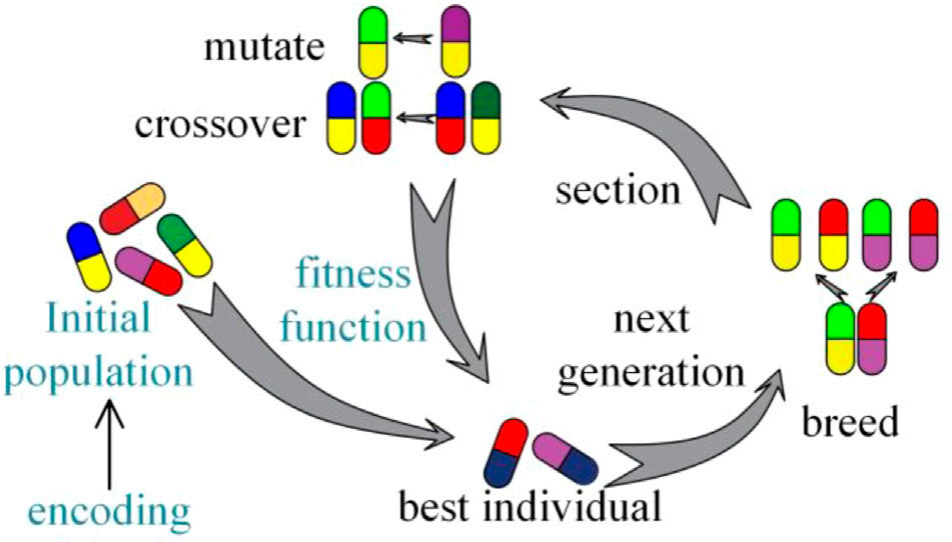
The process of genetic algorithm.

The process of genetic algorithms is shown in Figure 6. The crucial sections of genetic algorithms are fitness function, encoding, and initial population. Design parameters (*θ*, *R*, *d*) are encoded in a particular bit string, namely, “chromosomes.” Each chromosome corresponds to an individual and individuals form populations.

The main operations of genetic algorithms are selection, crossover, and mutation. The value of output power is regarded as the individual adaptability. Selection operation can determine whether chromosomes generate crossover and mutation according to fitness. There are many kinds of selection methods such as roulette, rank, and tournament. In this paper, we choose roulette to process selection operation. Roulette selection operator is expressed as

$$P_{i}=\frac{f_{i}}{\sum_{i=1}^{n}f_{i}}$$ (21)

where *p* is the probability that can be selected and *f* is the value of outlet power.

Crossover operation exchanges the fragments of nozzle structure parameter coding to form the next generation. Mutation changes one or more gene values in the coding of nozzle structural parameters. The strongest individuals are finally retained after several generations of elimination.

### Optimization Results of Curved-Tube Nozzle

**FIGURE 7:**
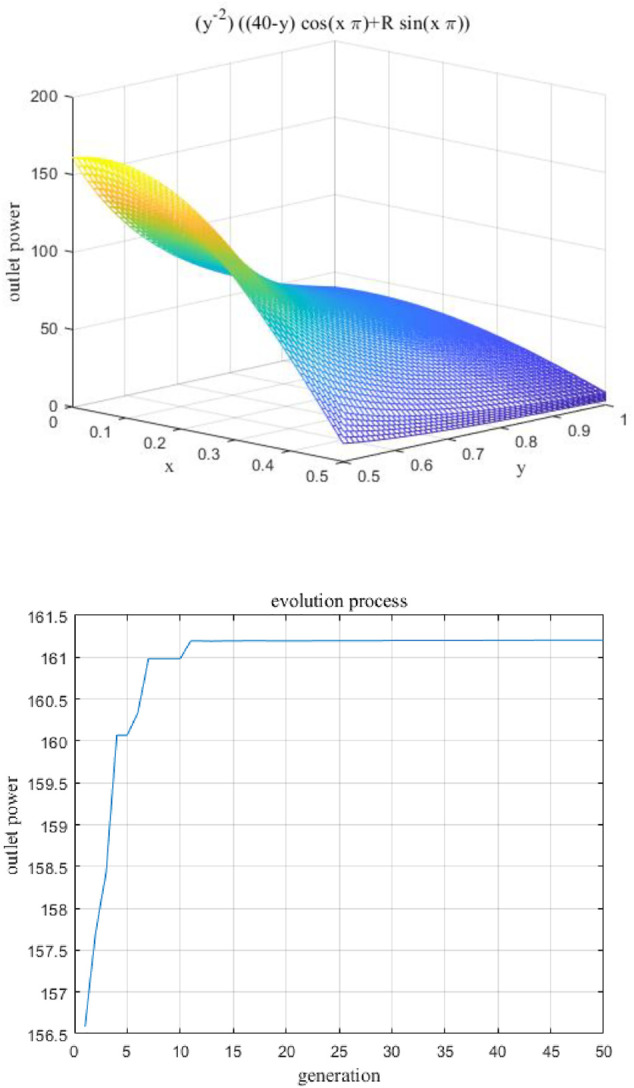
Optimization process diagram based on genetic algorithm. **(A)** Fitness function. **(B)** Calculation process of genetic algorithm.

It can be found from Figure 7A that structure parameters are non-linear and non-monotonic to the outlet power. The fitness function is theoretical model searching for the best combination of design parameters. It should be verified by numerical simulation and corresponding experiments of rotating spinning to avoid unreliable conclusion.

To explore the best structure of the curved-tube nozzle, the three structure parameters (*θ*, *R*, *d*) are optimized to maximize the objective function Eq. 21 based on the genetic algorithm. After multiple parameter adjustment and iterative operation, the basic setup properties of genetic algorithm are population size of 100 individuals, crossover probability 0.8, and 0.1 mutation rate. In Figure 7B, the calculation process of genetic algorithm can be seen. The maximum value of fitness function has been obtained after about 50 generations of evolution. The best design parameters corresponding to the maximum value of objective function 161.2 are shown in Table 4.

**TABLE 4:** Optimum nozzle structure parameter values

Parameters	Description	Value
Θ	Bending angle	10.8°
*R*	Curvature radius	8 mm
*d*	Nozzle diameter	0.5 mm

### Flow Field Simulation of Rotating Spinning

According to the dynamic model of the rotating spinning system, it can be seen that the flow field distribution is related to the bending angle, curvature radius, and nozzle diameter. Therefore, simulation experiments under different combinations of design parameters have been carried out by utilizing the finite-element CFD method.

### Model Establishment of Simulations of Spinning Solution

**FIGURE 8:**
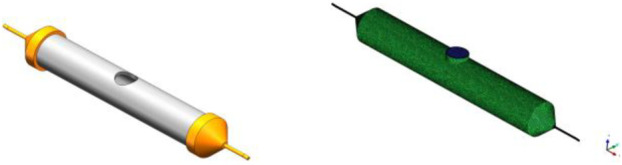
The spinneret model of curved-tube nozzle.

The three-dimensional motion model of the spinning solution is established as shown in Figure 8. The solid structure such as container wall and nozzle wall can be simplified by the fluid simulation software ICEM.

Nozzle outlet, solution inlet, nozzle wall, and tank wall are the four parts in the simulation model. The container diameter is 10 mm, the overall length is 60 mm, and the nozzle straight tube is 12 mm long. The unstructured grid division method is adopted, and the maximum grid size is 0.6 mm. The boundary layer is divided into four layers meshing as hexahedral with 0.01 mm initial height and 1.1 increase rate.

### Boundary Condition Setting for Rotating Spinning

The boundary conditions of rotating spinning motion model mainly include inlet boundary, outlet boundary, wall, dynamic mesh, and solution rheological parameters. The inlet boundary is velocity inlet, the hydraulic diameter is 6 mm, the outlet boundary is pressure outlet, and the hydraulic diameter is 2 mm.

The dynamic mesh is set as the rotating reference system, the rotating axis is *z* axis, and the rotating angular velocity is 4,000 rpm. The wall is set to move the wall relative to the grid area rotation speed of 0, and the rotation axis is *z* axis.

### Analysis for Simulation of Flow Field in Curved-Tube Nozzle

**FIGURE 9:**
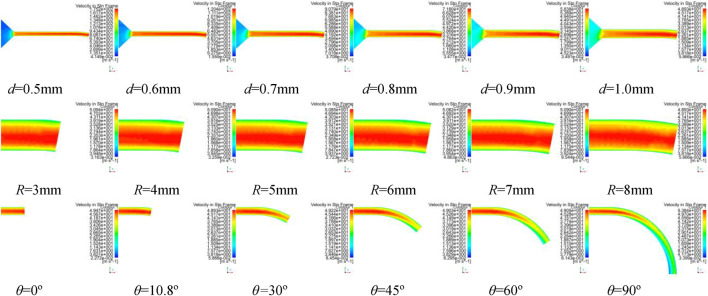
The velocity contours of spinning solution in tube of curved-tube nozzle.

Different simulations of solution motion are established with bending angle within 0–90°, curvature radius within 3∼8 mm, and nozzle diameter within 0.5∼1 mm. Figure 9–Figure 11 show simulation results of rotating spinning under different combinations of design structure parameters.

**FIGURE 10:**
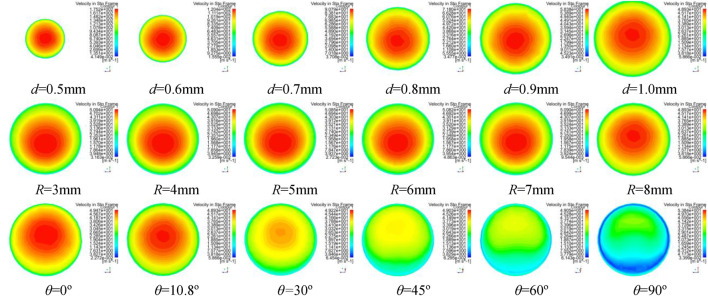
The velocity contours of spinning solution in tube of curved-tube nozzle.

**FIGURE 11:**
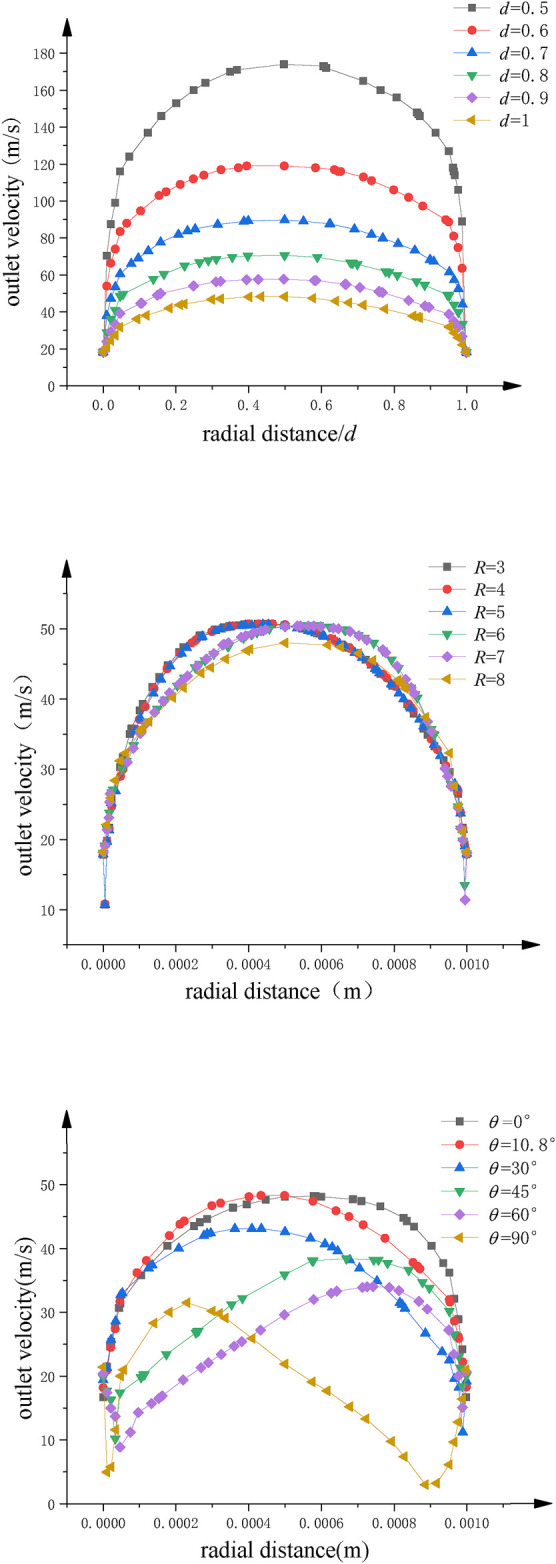
The velocity distribution of spinning solution at outlet of nozzle. **(A)** Nozzle diameter. **(B)** Curvature radius. **(C)** Bending angle.

Figure 9 and Figure 10 show the velocity contours of spinning solution in the nozzle tube and nozzle outlet, respectively. With the decrease of the nozzle diameter, the flow rate of the solution in the nozzle tube increases. This phenomenon shows that the relationship between nozzle diameter and compression effect is inversely proportional; the smaller the nozzle diameter, the better compression effect can be produced, resulting in more rapid flow velocity.

The maximum value of outlet velocity is concentrated at the tube axis when the bending angle of the nozzle is 10.8°. With the increase of bending angle, the flow field distribution gradually deviates from the tube axis and the outlet velocity gradually decreases. When the bending angle is 90°, the flow field in nozzle becomes chaotic and an obvious low-velocity region is produced, which reflects the negative influence of excessive bending angle on the flow field distribution.

Furthermore, the flow field distribution is more uniform when curvature radius is 8 mm. However, larger curvature radius consumes more solution kinetic energy, which leads to the decrease of the outlet velocity.

Figure 11 is a statistical analysis for the outlet velocity distribution along the radius direction. Figure 11A shows a significant linear relationship between the nozzle diameter and the outlet velocity. The larger the velocity, the greater the outlet velocity. Figure 11B and Figure 11C reflect the significant influence of curvature and bending angle on the deviation of flow field distribution in the rotating spinning process. Compared with nozzle diameter, these two parameters cannot increase the outlet velocity. However, the nozzle with the curvature of 8 mm and bending angle of 10.8° can reduce the deviation of velocity distribution at the outlet, make the flow field distribution more uniform, and benefit the stability of jet.

In conclusion, the best combination of structure parameters for curved-tube nozzle is bending angle 10.8°, curvature radius 8 mm, and nozzle diameter 0.5 mm, which is consistent with theoretical optimization results. It can effectively counteract flow field inhomogeneity and greatly improve the outlet velocity.

### Rotating Spinning Experiment

In the process of rotating spinning, angular velocity, solution rheological characteristics, structural parameters, and other factors will affect the final experimental results. To verify the theoretical optimization results, comparative experiments have been carried out with the same concentration PEO spinning solution and the same rotational speed. The electron microscopy has been applied to study the fiber diameter and morphology of nanofibers prepared by the ordinary straight nozzle and the curved-tube nozzle.

**FIGURE 12:**
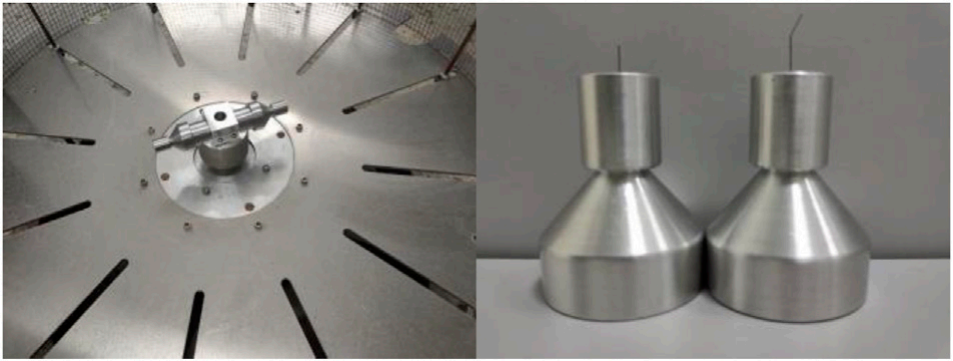
Experimental equipment diagram. **(A)** The rotating spinning equipment. **(B)** Curved and straight-tube nozzle.

The rotating spinning equipment and the nozzles used in the experiment are shown in Figure 12. The equipment can rotate at high speed by frequency conversion speed regulation, up to 6,000 rpm. We use two kinds of nozzle to prepare nanofibers: one is straight tube with nozzle diameter 0.5 mm; another is curved tube with bending angle 10.8°, curvature radius 8 mm, and nozzle diameter 0.5 mm. The rotating spinning experiment was carried out with 6% polyethylene oxide aqueous solution at the motor speed of 4,000 rpm.

**FIGURE 13:**
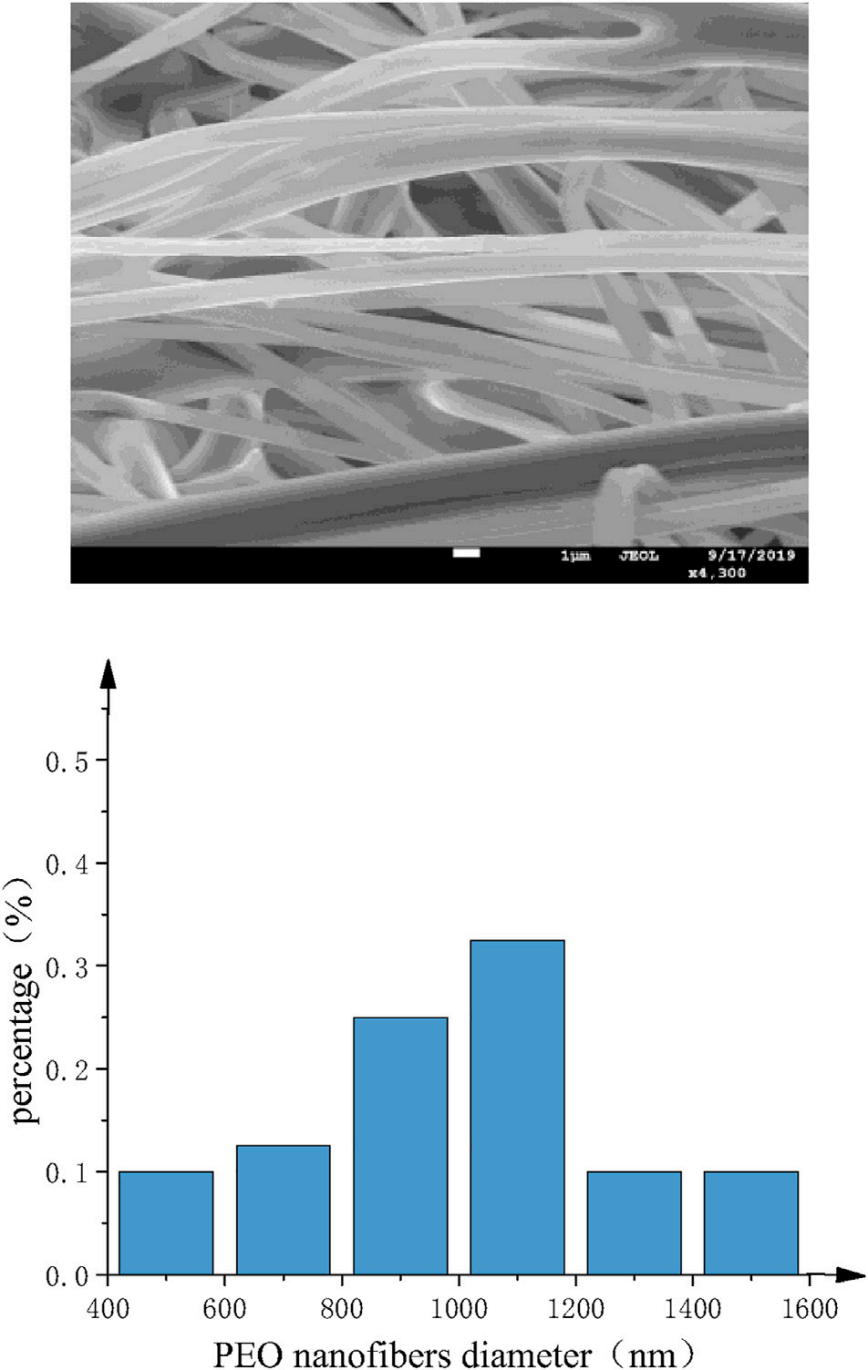
Straight-tube nozzle. (**A)** SEM images of PEO nanofibers. **(B)** Histogram of fiber diameter distribution.

**FIGURE 14:**
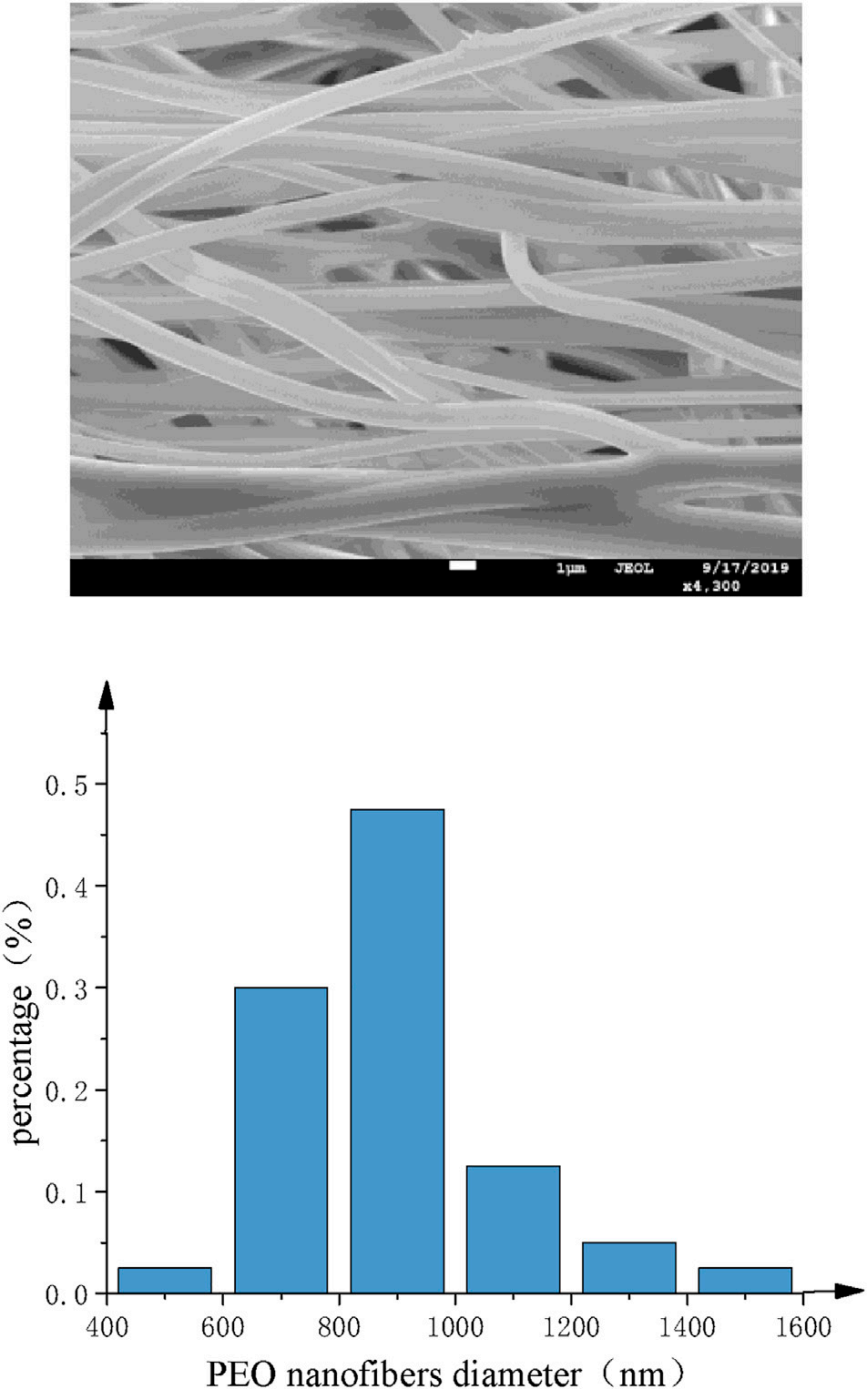
Curved-tube nozzle. **(A)** SEM images of PEO nanofibers. **(B)** Histogram of fiber diameter distribution.

SEM images of PEO nanofibers with different nozzles are shown as Figure 13 and Figure 14. It can be found that the diameter distribution of nanofibers prepared by straight nozzle is relatively dispersive in the range of 1,000∼1,200 nm. Also, the surface quality of nanofibers is poor. In comparison, the diameter of nanofibers prepared by curved-tube nozzle is mostly in the range of 800∼1,000 nm, the diameter distribution of nanofibers is more concentrated, and the morphology of nanofibers is more uniform. In conclusion, the overall quality of nanofibers prepared by curved nozzles has been greatly improved.

## Conclusion

In this paper, the motion and force of the spinning solution in container and nozzle during the rotating spinning process have been analyzed. Based on the genetic algorithm, the optimal structural parameters of the curved-tube nozzle are finally obtained, and the simulations and experiments are carried out for comparison and verification. It can be concluded that the curved-tube nozzle with bending angle 10.8°, curvature radius 8 mm, and nozzle diameter 0.5 mm can improve flow field distribution, increase outlet velocity, and fabricate high-quality nanofibers. However, the influences of friction resistance and gravity on spinning solution flow are not considered in the theoretical derivation, which leads to some differences between the simplified flow field distribution and simulation. Therefore, this problem would be considered more perfectly in the following research.

## Data Availability

The original contributions presented in the study are included in the article/Supplementary Material, further inquiries can be directed to the corresponding author.
