# Supplementary material for: Genetic Algorithm-Based Optimization of Curved-Tube Nozzle Parameters for Rotating Spinning
Source: Front Bioeng Biotechnol. 2021 Dec 3;9:781614. doi: 10.3389/fbioe.2021.781614 (PMC8678564; doi:10.3389/fbioe.2021.781614)
Supplement: Supplementary file 1 [file DataSheet1.docx]

Supplementary Material


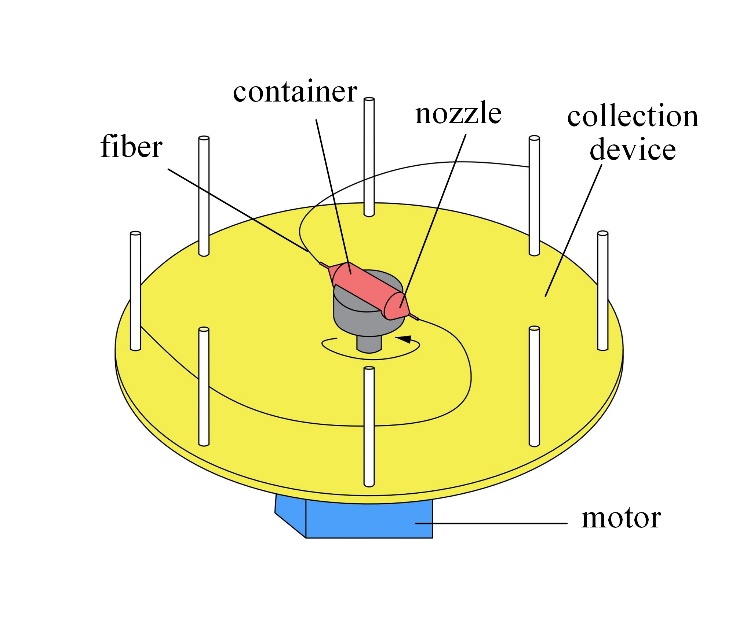


Figure 1 rotating spinning system


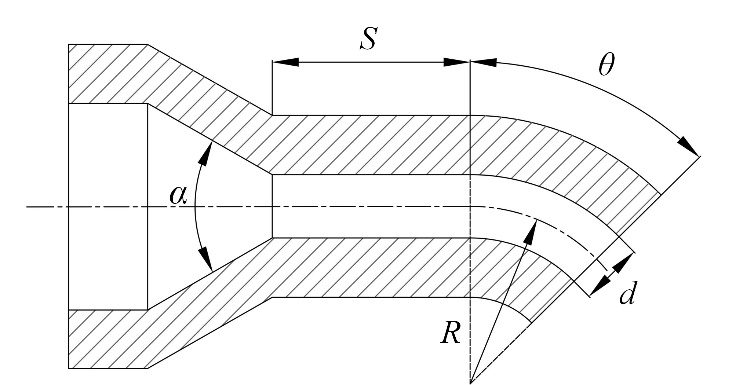


Figure 2 the structure of curved-tube nozzle


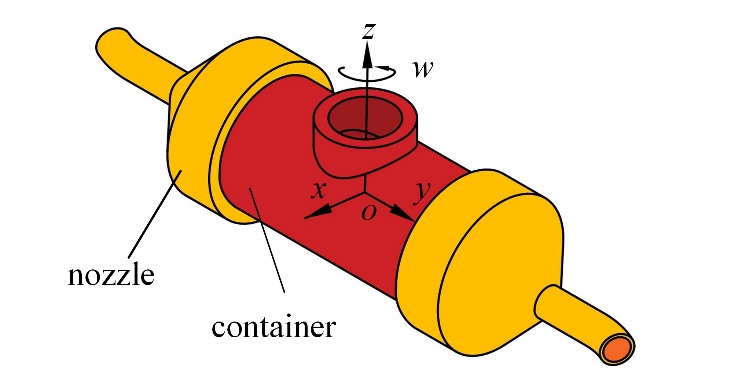

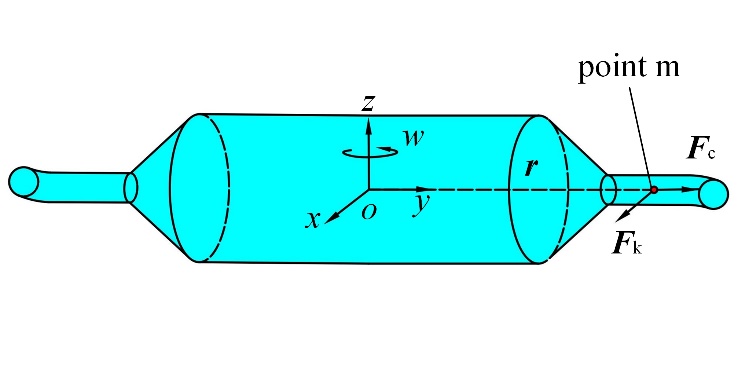


| (A) The structure of curved-tube nozzle and container | (B) The spinning solution in curved-tube nozzle and container |
| --- | --- |

Figure 3 motion model of rotating spinning


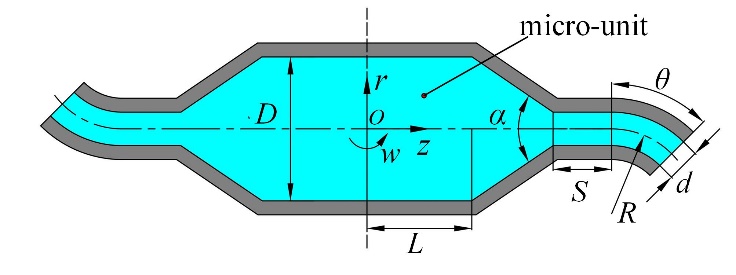


(A)one-dimensional flow of spinning solution


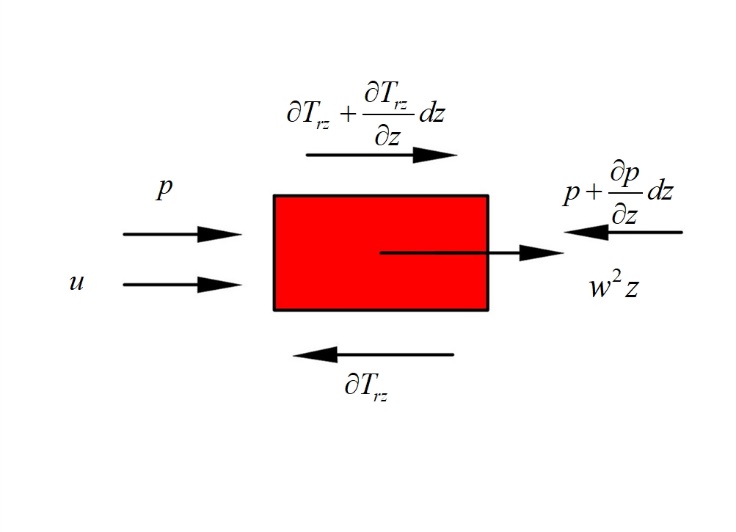


(B)Force diagram of micro-unit

Figure 4


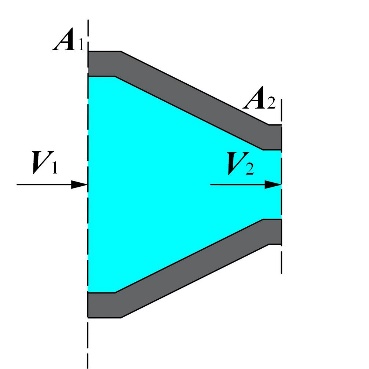

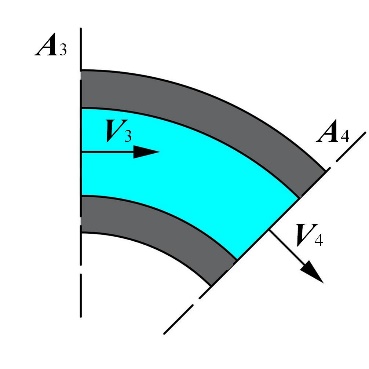


| (A) control body of Shrinkage tube | (B) control body of bend tube |
| --- | --- |

Figure 5 control body of the curved-tube nozzle


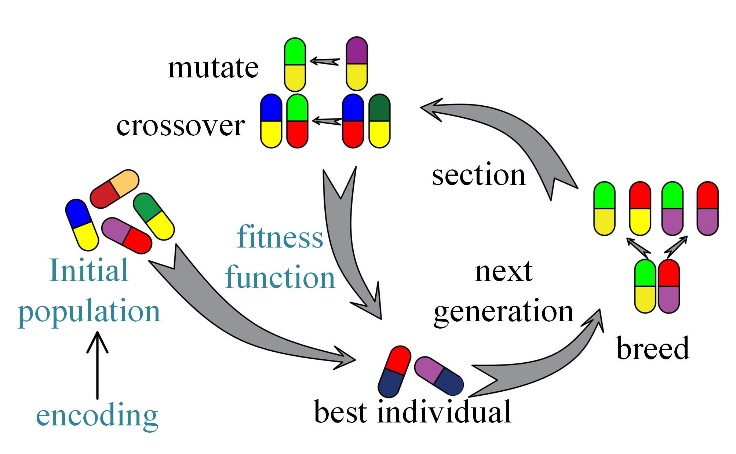


Figure 6 The process of Genetic Algorithm


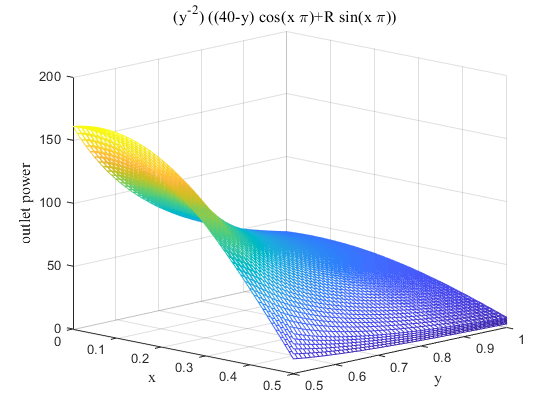


(A)Fitness function


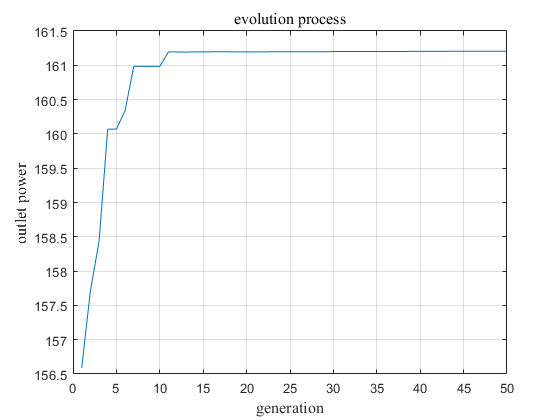


(B)Calculation Process of Genetic Algorithm

Figure 7




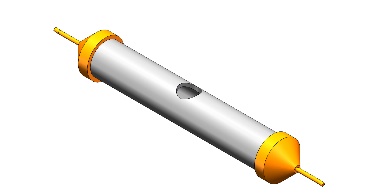


Figure 8 The spinneret model of curved-tube nozzle


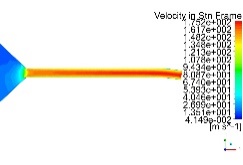

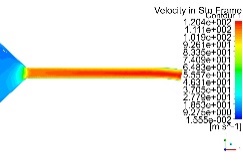

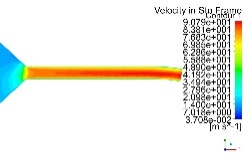

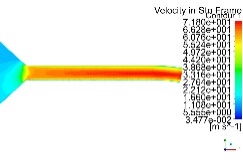

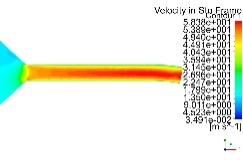

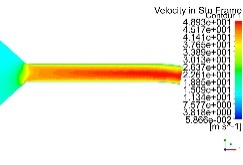


| *d*=0.5 | *d*=0.6 | *d*=0.7 | *d*=0.8 | *d*=0.9 | *d*=1.0 |
| --- | --- | --- | --- | --- | --- |


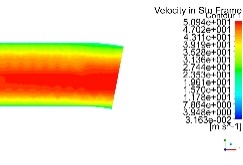

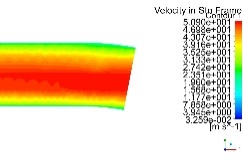

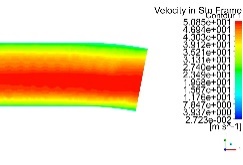

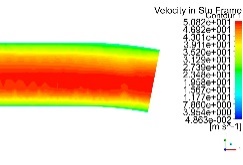

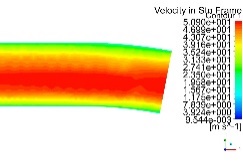

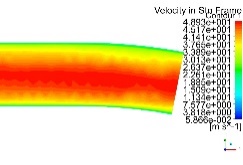


| *R*=3 | *R*=4 | *R*=5 | *R*=6 | *R*=7 | *R*=8 |
| --- | --- | --- | --- | --- | --- |


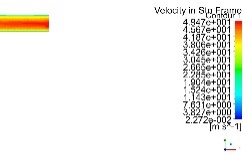

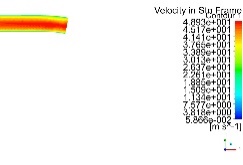

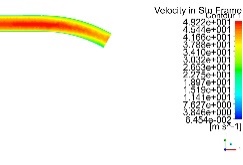

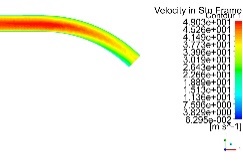

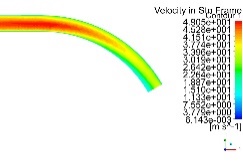

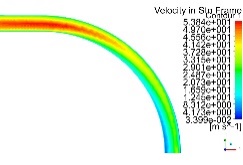


| θ=0º | θ=10.8º | θ=30º | θ=45º | θ=60º | θ=90º |
| --- | --- | --- | --- | --- | --- |

Figure 9 The velocity contours of spinning solution in tube of curved-tube nozzle


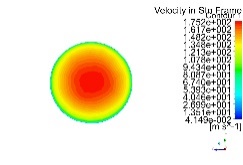

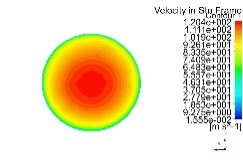

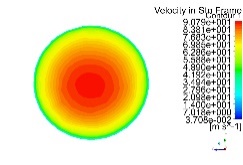

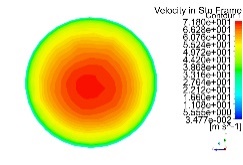

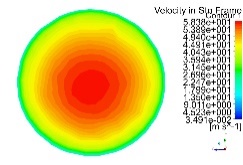

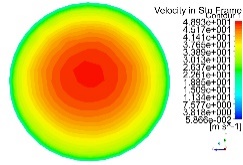


| *d*=0.5 | *d*=0.6 | *d*=0.7 | *d*=0.8 | *d*=0.9 | *d*=1.0 |
| --- | --- | --- | --- | --- | --- |


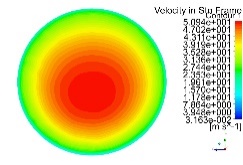

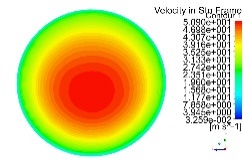

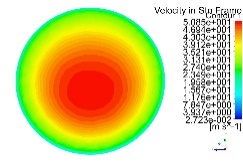

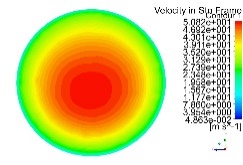

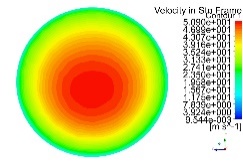

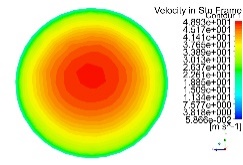


| *R*=3 | *R*=4 | *R*=5 | *R*=6 | *R*=7 | *R*=8 |
| --- | --- | --- | --- | --- | --- |


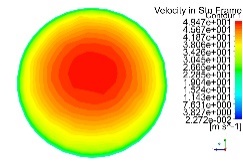

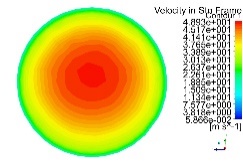

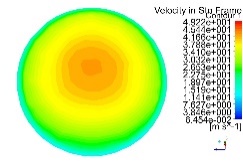

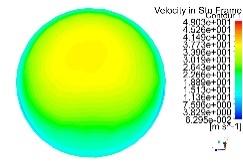

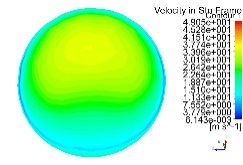

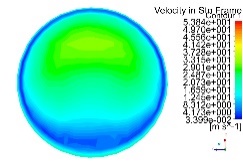


| θ=0º | θ=10.8º | θ=30º | θ=45º | θ=60º | θ=90º |
| --- | --- | --- | --- | --- | --- |

Figure10 The velocity contours of spinning solution at outlet of curved-tube nozzle

(A) Nozzle diameter

(B) Curvature radius

(C) Bending angle

Figure 11 The velocity distribution of spinning solution at outlet of nozzle


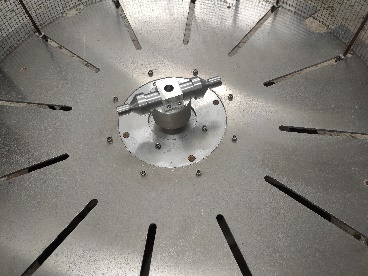

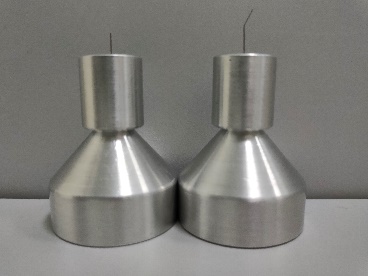


| (A) The rotating spinning equipment | (B) Curved and straight tube nozzle |
| --- | --- |

Figure 12





（A） straight-tube nozzle





(B) curved-tube nozzle

Figure 13 SEM images of PEO nanofibers at different nozzle

1. **Tables**

Table 1 System parameters of optimizing model

| item | Parameters | value |
| --- | --- | --- |
| optimization object | outlet power | max value |
| design Parameters | Bending angle *θ*  Curvature radius *R*  nozzle diameters *d* | [0,90]  [3,8]  [0.5,1] |
| other parameters | consistency index *k*  rheological index *n*  container diameter *D*  container length *L*  angular velocity *ω*  nozzle taper *α*  straight tube length *S*  density *ρ* | 15.3  0.464  10  30  4000  90  5  1000 |

Table 2 Optimum nozzle structure parameter values

| Bending angle | curvature radius | nozzle diameter |
| --- | --- | --- |
| 10.8º | 8mm | 0.5mm |
